# High frequency of enterotoxigenic *Bacteroides fragilis* and *Enterococcus faecalis* in the paraffin-embedded tissues of Iranian colorectal cancer patients

**DOI:** 10.1186/s12885-021-09110-x

**Published:** 2021-12-22

**Authors:** Nasibeh Khodaverdi, Habib Zeighami, Ahmad Jalilvand, Fakhri Haghi, Negar Hesami

**Affiliations:** 1 Department of Microbiology and Virology, School of Medicine, Zanjan University of Medical Sciences, Zanjan, Iran; 2 Department of Pathology, School of Medicine, Zanjan University of Medical Sciences, Zanjan, Iran; 3 Department of Microbiology, Zanjan Branch, Zanjan Islamic Azad University, Zanjan, Iran

**Keywords:** *Bacteroides fragilis*, Colorectal cancer, *Enterococcus faecalis*, *Fusobacterium spp*, *Streptococcus gallolyticus*

## Abstract

**Background:**

The association between specific bacteria and colorectal cancer (CRC) has been proposed. Only a few studies have, however, investigated this relationship directly in colorectal tissue with conflicting results. So, we aimed to quantitate *Streptococcus gallolyticus*, *Fusobacterium spp*, *Enterococcus faecalis* and enterotoxigenic *Bacteroides fragilis* (ETBF) in formalin-fixed and paraffin-embedded (FFPE) colorectal tissue samples of Iranian CRC patients and healthy controls.

**Methods:**

A total of 80 FFPE colorectal tissue samples of CRC patients (*n* = 40) and healthy controls (n = 40) were investigated for the presence and copy number of above bacterial species using quantitative PCR. Relative quantification was determined using ΔΔCT method and expressed as relative fold difference compared to reference gene.

**Results:**

Relative abundance and copy number of *E. faecalis* and ETBF were significantly higher in CRC samples compared to control group. *E. faecalis* was more prevalent than ETBF in tumor samples. Frequency of ETBF and *E. faecalis* in late stages (III/IV) of cancer was significantly higher than early stages (I/II). We did not detect a significant difference in abundance of *S. gallolyticus* and *Fusobacterium spp* between two groups.

**Conclusion:**

Our study revealed the higher concentration of *E. faecalis* and ETBF in FFPE samples of CRC patients than controls. However, additional investigations on fecal and fresh colorectal cancer tissue samples are required to substantiate this correlation.

## Background

Colorectal cancer (CRC) is one of the most common cancers worldwide, accounting for approximately 1.9 million new cases and 900.000 deaths in 2020 [1, 2]. The etiology of CRC is not fully understood. Several risk factors are associated with initiation and progression of CRC including genetic susceptibility, epigenetic and environmental factors such as diet, obesity, smoking, alcohol consumption and host immunity [1, 3, 4]. Because of high number of false positive cases in CRC screening systems such as Fecal Occult Blood Test (FOBT), identifying the sensitive biomarkers for early detection allows efficient treatment of CRC [1]. Previous studies have demonstrated a causal link between specific bacterial and viral pathogens and cancers such as gastric carcinoma, cervical cancer and hepatocellular carcinoma [3, 5–7]. A possible role of oncogenic bacteria in CRC development was first suggested in 1951 when a clinical association between *Streptococcus bovis* bacteremia/endocarditis and CRC was reported [1, 5, 6]. Since then, numerous studies have shown the enrichment of fecal or tissue samples of CRC patients with bacterial species such as enterotoxigenic *Bacteroides fragilis* (ETBF), *Fusobacterium nucleatum, Clostridium septicum*, *Enterococcus faecalis*, *Streptococcus gallolyticus,* Enteropathogenic *Escherichia coli* and *pks*^+^ *E. coli* [1, 4–6]. Several bacterial-induced carcinogenic mechanisms in CRC have been proposed including Wnt signaling activation, pro-inflammatory signaling and genotoxicity [5, 6]. Following bacterial infection and chronic inflammation, changes occur in the cellular microenvironment that leads to precancerous and finally cancerous events [2]. *pks*^+^ *E. coli* possesses a genotoxin colibactin, produced by the polyketide synthase genomic island (*pks*) that induces DNA damage, cell cycle arrest, mutations and chromosomal instability in eukaryotic cells. The interaction of LPS, FadA (Fusobacterium adhesion protein A) and Fap2 (Fusobacterium autotransporter protein 2) located on the surface of *F. nucleatum* with epithelial cell can affect host immune response via decreased apoptosis, cellular proliferation and DNA repair through activation of the nuclear factor-κB (NF-κB) and Wnt signaling pathways [3, 8]. The possible carcinogenic role of *S. gallolyticus* in CRC is mediated by overexpression of cyclooxygenase-2 (COX2), prevention of apoptosis and promotion of angiogenesis and inflammation. Furthermore, *E. faecalis* strains produce reactive oxygen species and extracellular superoxide anions that cause DNA damage and chromosomal instability [9]. A significant association between the presence of ETBF in fecal or colon biopsy specimens and CRC has been reported in previous studies. Secreted pro-inflammatory *B. fragilis* toxin (BFT) cleaves and degrades the extracellular domain of cell surface protein E-cadherin. Loss of E-cadherin triggers ß-catenin nuclear signaling, induces *c-myc* expression and IL-8 secretion [4, 10]. BFT also causes oxidative DNA damage and stimulates the high expression of IL-17. It is proposed that long-term colonization of colonic epithelial cells with ETBF may increase the risk of CRC [4, 10].

Although many studies have showed an association between the colorectal cancer development and intestinal microbiota, not all studies have yielded consistent results. Several studies demonstrated *F. nucleatum* as dominant bacteria in CRC patients, while others suggested *B. fragilis* as an abundant bacterium associated with CRC [3]. So, more research is needed to find association between specific bacteria and CRC.

Most studies investigating bacterial involvement in cancer development were based on fecal samples, while the composition of intestinal microbiota varies in the fecal and mucosal surface [1, 10]. Formalin-fixed and paraffin embedded (FFPE) archived tissues are extremely valuable sources for molecular diagnostic purposes in colorectal cancer [1, 11]. So, we used quantitative real-time polymerase chain reaction (qPCR) to investigate the presence and copy number of ETBF, *E. faecalis*, *S. gallolyticus* and *F. nucleatum* in FFPE colon tissue samples of colorectal cancer patients and healthy controls.

## Methods

### Sample collection

This case-control study was approved by the Research Ethics Committee of Zanjan University of Medical Sciences (IR.ZUMS.REC.1398.481). A total of 80 formalin-fixed, paraffin-embedded (FFPE) colorectal tissue samples from patients diagnosed with colorectal cancer (*n* = 40) and healthy controls (n = 40) were collected from archives of Pathology Department of Ayatollah Mousavi hospital in Zanjan Province, Iran. Colorectal tissue samples of CRC patients and control group were collected during colonoscopy. The control group underwent colonoscopy for various reasons and all of them were outpatients. No gastrointestinal disease was reported in control group and normal colon mucosa was confirmed. None of the cases or controls had a pre-colonoscopy chemo- or radiotherapy, previous history of other gastrointestinal diseases and antibiotic therapy within the past 1 month. The clinicopathological parameters including gender, age, family history of cancer, smoking, tumor stage and tumor location were collected according to medical records of patients. Sections of 10 μm in thickness from the top of FFPE tissue samples were prepared using a standard microtome with disposable blades and transported to the laboratory of Medical Microbiology. Then, normal and tumor tissues were dissected using sterile scalpels for DNA extraction.

### DNA extraction

DNA extraction from FFPE tissue samples was performed using GeneAll Exgene™ FFPE Tissue DNA kit (GeneAll Biotechnology, Songpa-gu, Seoul, South Korea). Three sections of 10 μm in thickness from FFPE samples was cut up and after deparaffinization, samples were incubated in 180 μl lysozyme (20 mg/ml) for 40 min at 37 °C. Then, 20 μl of proteinase K solution (20 mg/ml) was added and incubated at 56 °C for 1 h. The extraction steps were continued according to the manufacturer’s instructions. Also, extraction of DNA from non-tissue-containing paraffin sections was performed to assess any environmental bacterial contamination of blocks during fixation, embedding and processing.

The concentration and purity of DNA samples were determined using NanoDrop spectrophotometer (ND-1000, Nano-Drop Technologies, Wilmington, DE) at 260 and 260/280 nm, respectively. DNA samples were stored at − 20 °C for further analysis.

### Quantitative real-time polymerase chain reaction (qPCR)

Oligonucleotide primers targeted to detect conserved sequences specific for ETBF, *E. faecalis*, *S. gallolyticus* and *Fusobacterium spp* were selected from the literature and their specificity was confirmed by Primer BLAST (https://blast.ncbi.nlm.nih.gov/Blast.cgi) (Table 1). A conserved sequence present in all bacteria (universal primer) was also used as internal control in qPCR. First, the specificity of amplification was checked using conventional PCR and the amplicons were analyzed by agarose gel electrophoresis for specific band of amplified products. Then, PCR standardized conditions were used to carry out qPCR. Furthermore, conventional PCR was used to assess contamination of non-tissue-containing paraffin sections by universal primer.

**Table 1:** Primers used in this study

Bacterial pathogens	Target Gene	Primer sequence (5–3)	Amplicon (bp)	Annealing Temperature (°c)	Ref
***Fusobacterium spp***	*16 s ribosomal RNA*	CCCAAGCAAACGCGATAAGT GCGTTGCGTCGAATTAAACC	117	58	[1]
**ETBF**	*bft*	TGAAGTTAGTGCCCAGATGC CAGTAAAGCCTTCCAGTCC	150	58	[10]
***S. gallolyticus***	*SodA*	AAGCTGCGACAACTCGCTTT AAGCGTGTTCCCAAACGTCA	150	59	[1]
***E. faecalis***	*16 s ribosomal RNA*	CCCTTATTGTTAGTTGCCATCATT ACTCGTTGTACTTCCCATTGT	144	60	[12]
**Universal**	*16 s ribosomal RNA*	AAACTCAAAKGAATTGACGG CTCACRRCACGAGCTGAC	180	59	[13]

Standard curves were prepared for each run of qPCR using seven 10-fold dilutions of extracted DNA from reference strains of ETBF strain D-134, *E. faecalis* ATCC 29212*, S. gallolyticus* ATCC 49147 and *F. nucleatum* ATCC 25586 and DNA copy number was calculated using the following formula:

Number of copies = Mass (in grams) × 6.023× 10^23^/ Average mol. wt. of a base × template length. According to the standard curves, samples which did not have a fluorescent signal earlier than the Ct of 35 for *S. gallolyticus*, *Fusobacterium spp* and *E. faecalis* and 37 for ETBF were considered as negative.

Limit of detection (LOD) of primers was also determined using serial dilution of bacterial DNA. This was found to be approximately 10^2^ DNA copies for *B. fragilis* and 10 DNA copies for *S. gallolyticus*, *Fusobacterium spp* and *E. faecalis*.

Simplex qPCR was performed in a reaction mixture with total volume of 20 μl consisted of 10 μl of RealQ Plus 2x Master Mix Green High ROX™ (Ampliqon, Denmark), 0.4 μM of each the specific primer pairs, 30 ng of DNA in double distilled water. Assays were carried out in duplicate with an Applied Biosystems StepOnePlus™ Real-Time PCR System. Reported data are the mean values of duplicate qPCR analyses. Amplifications involved an initial denaturation at 95 °C for 10 min, followed by 40 cycles of denaturation at 95 °C for 30 s, annealing at 57–60 °C (Table 1) for 30 s, and extension at 72 °C for 30 s. Amplification specificity of each run was assessed by melting curve analysis. Relative quantification was also determined using the 2^-∆Ct^ method and expressed as relative fold difference compared to the reference gene (16S ribosomal RNA) conserved among all bacteria.

### Statistical analysis

Statistical analysis was performed with the Statistical Package for Social Sciences (SPSS), version 17.0 (SPSS, Inc., Chicago, IL). The data were presented as frequencies for qualitative variables and as means ± standard error mean (SEM) for quantitative variables. The Mann-Whitney U-test and Fisher’s exact tests were used to determine the significance of differences between two groups. *P* value of < 0.05 was considered significant.

## Results

The clinicopathological characteristics of patients are shown in Table 2. Briefly, a total of 40 FFPE colon tissue samples from CRC patients (19 males and 21 females) and 40 from healthy control cases (22 males and 18 females) were investigated. The mean age of CRC patients was 56.37 years (range 31–86 years) and for healthy controls was 60 years (range 20–82). The majority of CRC patients were stage III or IV cancer (65%), while stages I and II were 10 and 25%, respectively. In CRC patients, 62.5% of tumors were located in distal colon and 37.5% were in proximal. The distal colon includes the descending colon and the sigmoid colon and proximal colon includes the cecum, ascending colon and the transverse colon.

**Table 2:** Clinicopathological characteristics of patients

	CRC (***n*** = 40)	Control (***n*** = 40)
Male	19 (47.5%)	22 (55%)
Female	21 (52.5%)	18 (45%)
Age (Mean ± SE)	56.37 ± 14.7	60 ± 15.11
Tumor location		
Distal colon	25 (62.5%)	–
Proximal colon	15 (37.5%)	–
Tumor stages		
I	4 (10%)	–
II	10 (25%)	–
III	11 (27.5%)	–
IV	15 (37.5%)	–
Smoking	15 (37.5%)	17 (42.5%)
Family history	4 (10%)	3 (7.5%)

16S ribosomal RNA gene was not detected in non-tissue-containing paraffin sections. To determine whether CRC was associated with ETBF, *S. gallolyticus*, *Fusobacterium spp* and *E. faecalis*, we used qPCR to compare the frequency and quantity of the bacteria in colorectal tumors and normal colon tissue. Log_10_ values [means ± standard deviation (SD)] of DNA copy numbers were calculated for each bacterium. The number of positive samples and the copy numbers of each bacterium in normal and tumor tissues are presented in Table 3. Relative quantification was also determined by the 2^-∆Ct^ method and is shown in Table 4. *E. faecalis* was detected in 85% of CRC samples and ETBF in 45% of cases. The presence of *E. faecalis* and ETBF was higher in tumor samples compared to control group (*P* < 0.05). *E. faecalis* was more prevalent than ETBF in tumor samples (*P* < 0.05). For ETBF, log_10_ copies DNA ml^− 1^ were 3.8 and 2.7 for CRC samples and control group, respectively (*P* = 0.003). As shown in Table 3, higher concentration of *E. faecalis* was found in CRC patients than control group (*P* ≤ 0.05). The presence of ETBF and *E. faecalis* was not comparable when stratifying tumor samples based on cancer stages and cancer location (Tables 5 and 6).

**Table 3:** Presence, copy number and Ct values of bacteria in normal and tumor tissues

	CRC (n = 40)	Control (***n*** = 40)	***P*** value
**Qualitative presentation, percent patients positive** ^**a**^			
***Fusobacterium spp***			
Yes	27 (67.5%)	24 (60%)	0.48
No	13 (32.5%)	16 (40%)	
**ETBF**			
Yes	18 (45%)	6 (15%)	0.003
No	22 (55%)	34 (85%)	
***S. gallolyticus***			
Yes	29 (72.5%)	28 (70%)	0.80
No	11 (27.5%)	12 (30%)	
***E. faecalis***			
Yes	34 (85%)	25 (62.5%)	0.005
No	6 (15%)	15 (37.5%)	
**Range and median values of Ct**			
*Fusobacterium spp*	25.8–38.3 (30.7)	25.3–39.1 (31.3)	> 0.05
ETBF	23.4–38.2 (37.5)	26.9–39.1 (38.8)	> 0.05
*S. gallolyticus*	26.4–36.8 (31.2)	28.1–37.8 (31.8)	> 0.05
*E. faecalis*	21.5–37 (28.3)	23.3–38.1 (29.7)	> 0.05
**Log copies DNA ml**^**−1 b**^			
*Fusobacterium spp*	2.9 ± 0.32	2.8 ± 0.15	0.23
ETBF	3.81 ± 0.07	2.7 ± 0.63	0.002
*S. gallolyticus*	3.04 ± 0.12	2.98 ± 0.31	0.39
*E. faecalis*	4.4 ± 0.10	3.1 ± 0.43	0.001

^a^Values noted as number (percentage), Fisher’s exact test

^b^Values noted as log copies DNA ml^−1^, Mann–Whitney test

**Table 4:** Quantitation of intestinal bacteria in CRC and controls. Quantitative values are shown as relative difference, calculated by the 2^-∆Ct^ method and expressed as relative fold difference compared to the reference gene. Values shown are mean + SEM

	CRC (n = 40)	Control (n = 40)	***P*** value
**Relative difference**			
*Fusobacterium spp*	0.03 ± 0.04	0.02 ± 0.03	0.62
ETBF	0.03 ± 0.01	0.003 ± 0.02	0.025
*S. gallolyticus*	0.04 ± 0.01	0.02 ± 0.02	0.18
*E. faecalis*	0.05 ± 0.03	0.01 ± 0.02	0.029

**Table 5:** Presence of bacteria in CRC patients with respect to cancer stage

Stage of cancer	Stage I (n = 4)	Stage II (*n* = 10)	Stage III (*n* = 11)	Stage IV (*n* = 15)
Bacteria				
***Fusobacterium spp*** **(*n*** **= 27)**	3 (75%)	5 (50%)	8 (72.7%)	11 (73.3%)
**ETBF (*n*** **= 18)**	2 (50%)	4 (40%)	5 (45.4%)	7 (46.6%)
***S. gallolyticus*** **(*n*** **= 29)**	4 (100%)	5 (50%)	11 (100%)	9 (60%)
***E. faecalis*** **(*n*** **= 34)**	4 (100%)	7 (70%)	11 (100%)	12 (80%)

**Table 6:** Presence of bacteria in CRC patients with respect to cancer location

Tumor location	Proximal colon (n = 15)	Distal colon (*n* = 25)
Bacteria		
***Fusobacterium spp*** **(n = 27)**	9 (60%)	18 (72%)
**ETBF (n = 18)**	7 (46.6%)	11 (44%)
***S. gallolyticus*** **(n = 29)**	10 (66.6%)	19 (76%)
***E. faecalis*** **(n = 34)**	12 (80%)	22 (88%)

According to results, we did not detect a significant difference in the frequency and DNA copy number of *S. gallolyticus* and *Fusobacterium spp* between two groups (Tables 3 and 4).

## Discussion

In recent years, several reports suggest that intestinal microbial dysbiosis may be an etiological factor in the initiation and progression of colorectal cancer [1, 2, 14, 15]. Although the enrichment of fecal or tissue samples of CRC patients with bacterial species such as ETBF, *F. nucleatum, E. faecalis*, *S. gallolyticus* and EPEC has been shown in previous studies, not all reports have consistent results [1, 2, 4–6]. Furthermore, data on CRC-associated microbiota in Iranian populations are scarce. Therefore, in order to find a better understanding of this association, we used qPCR to investigate the presence and copy number of ETBF, *E. faecalis*, *S. gallolyticus* and *F. nucleatum* in FFPE colon tissue samples of Iranian CRC patients and healthy controls. According to our results, relative abundance and copy number of *E. faecalis* and ETBF were significantly higher in CRC samples compared to control group. *E. faecalis* was more prevalent than ETBF in tumor samples. In our study, we used the 16 s ribosomal RNA gene as an internal control, but it was better to use a reference gene such as prostaglandin transporter (PGT) for normalization. Several case-control studies have reported that the abundance of ETBF was higher in ulcerative colitis, colorectal adenoma and CRC patients than controls [3–6, 9, 10]. In study conducted by Rezasoltani et al. (2018), higher numbers of ETBF, *E. faecalis, F. nucleatum, S. bovis*, and *Porphyromonas* spp. were reported in adenomatous polyp patients in contrast to controls [9]. In our previous study, we demonstrated the association between fecal ETBF and CRC and suggested that fecal detection of ETBF may be a potential biomarker for colorectal cancer diagnosis [4]. Furthermore, Shariati et al. (2021) showed a significantly higher abundance of *B. fragilis* and *F. nucleatum* in fresh frozen biopsies of colorectal lesions of Iranian CRC patients compared to adjacent normal mucosal tissues. However, they could not detect such a relation for *S. gallolyticus* and EPEC [16].

*Enterococcus faecalis* as one of the most common Gram-positive cocci in human stools produces extracellular superoxide, hydrogen peroxide and hydroxyl radicals which cause DNA damage in mammalian cells and chromosomal instability that lead to colorectal cancer [17]. We detected higher number of *E. faecalis* in CRC patients compared to control group. In consistent with our results, Balamurugan et al. (2008) demonstrated higher level of *E. faecalis* in stool of CRC patients compared to healthy volunteers. According to their results, population of *Eubacterium rectale* and *Faecalibacterium prausnitzii* was decreased approximately fourfold in CRC patients compared to healthy group. These changes in bacterial population in colon could potentially lead to epithelial cell damage and increased turnover and may be an etiological factor of CRC [17]. Furthermore, in study conducted by Zhou et al. (2016), the median abundance of *Fusobacterium* spp., *E. faecalis* and ETBF in tumor tissues was significantly higher than adjacent normal tissue and healthy controls [6]. However, in a prospective case cohort study of consecutive colonoscopy patients, fecal *E. faecalis* population was identified as unstable over > 1 year and an association between superoxide-producing *E. faecalis* and large colon adenomas or cancer was not found [18].

According to our results, the presence of ETBF and *E. faecalis* was not comparable when stratifying tumor samples based on cancer stages and cancer location.

Despite the noted association with CRC, we did not detect a significant difference in the frequency and copy number of *S. gallolyticus* and *Fusobacterium spp* between CRC patients and controls. The association between *S. gallolyticus* endocarditis/bacteremia and CRC is well established [19]. However, none of our patients had a history of bacteremia/bacterial-endocarditis and this may explain the lack of difference in abundance of *S. gallolyticus* between two groups. In agreement with our results, Mahmoudvand et al. (2017) reported no association between *S. gallolyticus* and colorectal cancer in paraffin-embedded biopsy specimens [20]. Only in studies conducted by Abdulamir et al. (2010) and Farajzadeh Sheikh et al. (2020), *S. gallolyticus* was detected with higher frequency in CRC patients with and without a history of bacterial endocarditis/bacteremia compared to healthy controls [21, 22]. According to Bundgaard-Nielsen et al. (2019) and Viljoen et al. (2015) studies, *S. gallolyticus* was not detected in any of colorectal tissue samples. Their findings could potentially be explained through ethnic differences in susceptibility to colorectal colonization of *S. gallolyticus* or geographical differences in *S. gallolyticus* distribution. Furthermore, these discrepancies may also be related to application of diverse specimens (like fresh frozen tissues, FFPE and stool) and different detection methods (such as qPCR, pyrosequencing, Fluorescence In Situ Hybridization) used for detection of *S. gallolyticus* [1, 5, 16].

In contrast to previous studies [5, 6, 9, 23], we did not observe a difference in abundance of *Fusobacterium spp* between CRC patients and controls. Furthermore, Bundgaard-Nielsen et al. (2019) showed that *F. nucleatum* was distributed equally in tumors, paired normal tissue and diverticula, but significantly less present in adenomas [1]. However, *F. nucleatum* abundance in CRC patients varied between 13 and 87% in different countries [5, 6, 9, 16, 23, 24]. According to Shariati et al. (2021) and Kashani et al. (2020) from Iran, *F. nucleatum* was highly abundant in CRC tissues (23 and 68%, respectively) compared to adjacent normal mucosa [16, 24]. Environmental factors such as weight, body mass index, diet, and geographical location may play an active role in bringing about this variation [16].

Our study also has several limitations. One of the limitations was the small number of included samples and the use of formalin fixed colorectal tissue specimens. Since formalin fixation causes cross-linking of DNA-tissue protein, which can prevent amplification. Also, DNA fragmentation may occur in formalin fixed tissue, which may limit our ability to detect bacteria [1, 11, 25]. Because all FFPE samples were handled similarly, we do not expect the formalin fixation to affect the observed differences in bacterial load and prevalence between diagnoses. On the other hand, we had a specific focus on the bacterial species *E. faecalis*, ETBF, *S. gallolyticus* and *Fusobacterium* spp. Further studies are need to investigate a potential role of other bacterial species as *E. coli* and *C. septicum*, *Prevotella* and *Acinetobacter* in CRC.

## Conclusions

Our study revealed the higher concentration of *E. faecalis* and ETBF in FFPE samples of CRC patients than controls. However, additional investigations on fecal and fresh colorectal cancer tissue samples are required to substantiate this correlation.

## Data Availability

All data generated or analyzed during this study are included in the manuscript.

## Abbreviations

CRC: Colorectal cancer
ETBF: Enterotoxigenic *Bacteroides fragilis*
FFPE: Formalin-fixed and paraffin-embedded
FOBT: Fecal Occult Blood Test
NF-κB: Nuclear factor-κB
COX2: Cyclooxygenase − 2
BFT: *B. fragilis* toxin.
